# Role of integrin αvβ5 receptor and microglia in delayed-onset muscle soreness in mice

**DOI:** 10.1007/s10974-026-09742-1

**Published:** 2026-10-23

**Authors:** Bruno Lopes Gonçalves Rocha, Flavio Protasio Veras, Rafaela Silva dos Santos, Albená Nunes-Silva, Thiago Roberto Lima Romero, Giovane Galdino

**Affiliations:** 1 Laboratory of Neuroimmunobiology of Pain, Center for Experimental Biology, Federal University of Alfenas, Alfenas, 37131-001 Minas Gerais Brazil; 2 Inflammation and Exercise Immunology Laboratory (LABIIEX), Federal University of Ouro Preto Sports Center, Ouro Preto, 35400-000 MG Brazil; 3 Laboratory of Pain and Analgesia, Pharmacology Department, Federal University of Minas Gerais, Belo Horizonte, 31270-901 Minas Gerais Brazil

**Keywords:** Irisin, αvβ5 receptor, Nociception, Aerobic exercise

## Abstract

Delayed-onset muscle soreness (DOMS) is characterized by muscle pain, stiffness, and tenderness following intense or unaccustomed exercise. In humans, symptoms typically arise 6–72 h post-exercise. This study investigated a novel mechanism underlying DOMS, focusing on the involvement of the irisin receptor integrin αvβ5 and spinal microglia in its development. Male C57BL/6 mice were subjected to a DOMS model induced by an acute aerobic exercise protocol, and nociceptive thresholds were assessed using the von Frey filament test. The role of spinal integrin αvβ5 was evaluated via intrathecal administration of the antagonist cilengitide, given both prior to exercise and 24 h post-exercise. To assess irisin’s nociceptive effects, it was also administered intrathecally. Additionally, minocycline, a microglial inhibitor, was administered before irisin to determine microglial involvement. An in vitro assay evaluated microglial activation by irisin via flow cytometry. Pro- and anti-inflammatory cytokines were quantified by ELISA. Cilengitide reversed exercise-induced DOMS and reduced spinal pro-inflammatory cytokines. Irisin induced nociception, which was attenuated by minocycline. Irisin also activated microglia in vitro, increasing pro-inflammatory cytokine production. These findings suggest integrin αvβ5 and spinal microglia play critical roles in DOMS pathogenesis, with irisin and pro-inflammatory cytokines as key mediators.

## Introduction

Delayed-onset muscle soreness (DOMS) is characterized by a decreased pain threshold in the affected musculature, resulting in discomfort or pain following strenuous or unaccustomed physical activity (Wilke and Behringer 2021). In humans, symptoms typically develop within 6–72 h post-exercise, peaking between 24 and 48 h and persisting for up to 4–5 days, although in some cases they may last up to 7 days (Peake et al. 2017). In murine models, however, the time course is generally shorter, with nociceptive responses emerging earlier, peaking around 24–48 h, and resolving within approximately 72 h depending on the experimental protocol (Peake et al. 2017). The underlying mechanism of DOMS is believed to involve an inflammatory response triggered by microtrauma to the contractile elements of muscle tissue (Sonkodi et al. 2020). This damage promotes the release and accumulation of inflammatory mediators, including histamine, neutrophils, bradykinin, and prostaglandins, which contribute to the sensitization of group III and IV sensory afferent nerve endings in skeletal muscle. This peripheral sensitization is considered a key factor in the development of exercise-induced muscle pain (Sonkodi et al. 2020).

In addition to classical inflammatory mediators, recent studies have identified alternative mechanisms involved in the pathophysiology of DOMS. Dos Santos et al. (2020) reported that heat shock protein 70 (HSP70), released by skeletal muscle following exercise, may activate Toll-like receptor 4 (TLR4) in the spinal cord, thereby contributing to nociceptive synaptic transmission during DOMS. Sonkodi (2025) proposed that an acquired channelopathy of the mechanosensitive Piezo2 ion channel in primary afferent neurons may disrupt the function of the voltage-gated sodium channel Nav1.1, promoting the development of DOMS. Furthermore, a review by Mizumura and Taguchi (2024) highlighted the involvement of several additional molecular mediators, including nerve growth factor (NGF), cyclooxygenase-2 (COX-2), glial cell line-derived neurotrophic factor (GDNF), transient receptor potential channels TRPV1 and TRPV4, and acid-sensing ion channels (ASICs). Despite these emerging insights, the underlying mechanisms of DOMS remain incompletely understood, particularly in comparison to other well-characterized pain conditions.

Myokines are peptides released by skeletal muscle in response to contraction, injury, or inflammation and act as key mediators of muscle–organ crosstalk (Lee and Jun 2019; Chen et al. 2021). Among these, irisin has emerged as a particularly relevant exercise-induced myokine due to its pleiotropic effects on metabolism, tissue repair, and central nervous system function (Barbalho et al. 2020a, b a). Irisin is generated by proteolytic cleavage of FNDC5 and is rapidly released into the circulation during physical activity, where it can modulate inflammatory responses, cellular stress pathways, and inter-tissue communication (Pedersen et al. 2007; Barbalho et al. 2020a, b b). Importantly, recent evidence suggests that irisin may play a role in muscle regeneration and in the modulation of pain-related signaling, including neuroinflammatory processes that contribute to nociceptive sensitization. These properties position irisin as a potential link between muscle activity and the development of delayed onset muscle soreness (DOMS). Despite this, the specific contribution of irisin to DOMS and its underlying mechanisms remains poorly understood.

In this context, several inflammatory mediators, including myokines, have the potential to induce pain by activating central nociceptive pathways. Specifically, these mediators can modulate the nociceptive synapse between primary afferent neurons and second-order neurons in the dorsal horn of the spinal cord (Pedersen et al. 2007). This process is further amplified by the activation of glial cells—particularly microglia—which release pro-inflammatory and pronociceptive cytokines (Gzerani, 2021). Therefore, given its regulatory effects on inflammation and neural function, irisin emerges as a potential mediator linking muscle activity to central nociceptive processing. Based on this rationale, the present study aimed to investigate the role of irisin and its receptor, integrin αvβ5, as well as the involvement of microglia, in the development of DOMS at the spinal level.

## Materials and methods

### Animals

All experiments were conducted using male C57BL/6 wild-type mice (7–8 weeks old), obtained from the vivarium of the Federal University of Alfenas. Animals were housed in groups of six per cage, with ad libitum access to food and water. They were maintained under controlled conditions, with a 12-hour light/dark cycle, temperature ranging between 22 and 24 °C, and relative humidity of 50 ± 5%. The study was approved by the Ethics Committee for the Use of Animals of the Federal University of Alfenas (protocol number 0046/2022) and was conducted in accordance with the guidelines of the International Association for the Study of Pain (IASP) for the use of animals in research (Zimmermann 1983).

### Substances used in the study

In this study, the following substances were used: cilengitide, a selective antagonist of the integrin αvβ5 receptor for irisin (Sigma-Aldrich, USA; #SML1594); recombinant irisin (Sigma-Aldrich, USA; #SRP6542); and minocycline, a microglial inhibitor (Sigma-Aldrich, USA; #PHR1801). All substances were diluted in sterile saline (0.9%). The doses of each substance were selected and adjusted according to previously published studies (Danemi et al., 2018; Mishra et al. 2020; Tu et al. 2022).

### Intrathecal injections

For intrathecal administration, animals were anesthetized with 2% isoflurane and placed in a prone position. Each substance was injected into the intrathecal space at the L5–L6 intervertebral segment, using a 10 µl syringe to deliver a volume of 5 µl, following the method described by Hylden and Wilcox (1980).

### Acute aerobic exercise protocol

The DOMS model employed in this study was adapted from Dos Santos et al. (2020). One day prior to the exercise protocol, all animals were acclimated to an electric treadmill designed for mice (Ugo Basile, Italy) by walking for five minutes at a low speed of 5 m/min. On the following day, the mice underwent a progressive running protocol, starting at 5 m/min for the first five minutes, increasing to 8.5 m/min until the tenth minute, then to 10.7 m/min until the fifteenth minute, and finally maintaining 13.4 m/min until the end of the session, with no incline. No external stimuli were applied to encourage running during the protocol. Animals that voluntarily ceased running were promptly removed from the treadmill and returned to their home cages to avoid excessive stress; all animals were retained in the experimental groups for subsequent analyses. Control animals remained on the stationary treadmill for an equivalent average duration to that of the exercised group. All protocols were conducted between 9:00 and 11:00 AM.

### Plantar mechanical sensitivity measurement

Plantar mechanical sensitivity was assessed using calibrated von Frey monofilaments (Aesthesio, USA). Animals were acclimated for 30 min in dark glass boxes placed on a metal grid platform, allowing access to the plantar surface of the right hind paw. Monofilaments with target forces of 0.07, 0.16, 0.4, 0.6, 1.0, 1.4, and 2.0 g, corresponding to evaluator sizes 2.83, 3.22, 3.61, 3.84, 4.08, 4.17, and 4.31, respectively, were sequentially applied to the plantar surface of the paw. The mean force corresponding to three withdrawal responses was recorded as the paw withdrawal threshold (Elisei et al. 2020). This procedure was used to assess exercise-induced plantar mechanical hypersensitivity and was not interpreted as a direct measurement of deep-muscle mechanical hyperalgesia.

### Experimental protocol

Baseline measurements of the nociceptive threshold were initially obtained. Subsequently, animals were randomly assigned to one of two experimental groups: the Exercise Group (*n* = 6), which underwent the DOMS protocol, and the Control Group (*n* = 6), which consisted of non-exercised animals. The nociceptive threshold was reassessed at 24 and 48 h post-protocol. Experiments were independently repeated using separate cohorts to ensure reproducibility of the findings.

To evaluate the involvement of the irisin receptor αvβ5, the antagonist cilengitide was administered intrathecally prior to exercise. Animals were divided into the following groups (*n* = 6 per group): (I) Exercise + cilengitide 10 nmol: exercised animals pre-treated with 10 nmol of cilengitide; (II) Exercise + cilengitide 100 nmol: exercised animals pre-treated with 100 nmol of cilengitide; (III) Exercise + saline: exercised animals pre-treated with saline, the vehicle for cilengitide; (IV) Non-exercise + cilengitide 100 nmol: non-exercised animals pre-treated with 100 nmol of cilengitide; (V) Non-exercise + saline: non-exercised animals treated with saline, the vehicle for cilengitide. Similar groups to those described above were used to investigate the involvement of the αvβ5 receptor in the maintenance of DOMS, with cilengitide (100 nmol, intrathecally) administered 24 h after exercise.

Furthermore, to evaluate the potential nociceptive effect of irisin in DOMS, recombinant irisin was administered intrathecally, and the nociceptive threshold was assessed at 24 and 48 h post-administration. The experimental groups included: (I) Irisin group, in which animals received 250 ng of recombinant irisin; (II) Saline group, in which animals received saline, the vehicle for irisin.

To investigate whether irisin promotes nociception through glial cell activation, an experiment was conducted in which minocycline, a microglial inhibitor, was administered 10 min prior to irisin. The following groups were included (*n* = 6 per group): (I) Minocycline + irisin group: animals received minocycline (100 µg) followed by irisin (250 ng); (II) Irisin group: animals received irisin alone (250 ng); (III) Saline group: animals received two administrations of saline as vehicle controls.

**Fig. 1:**
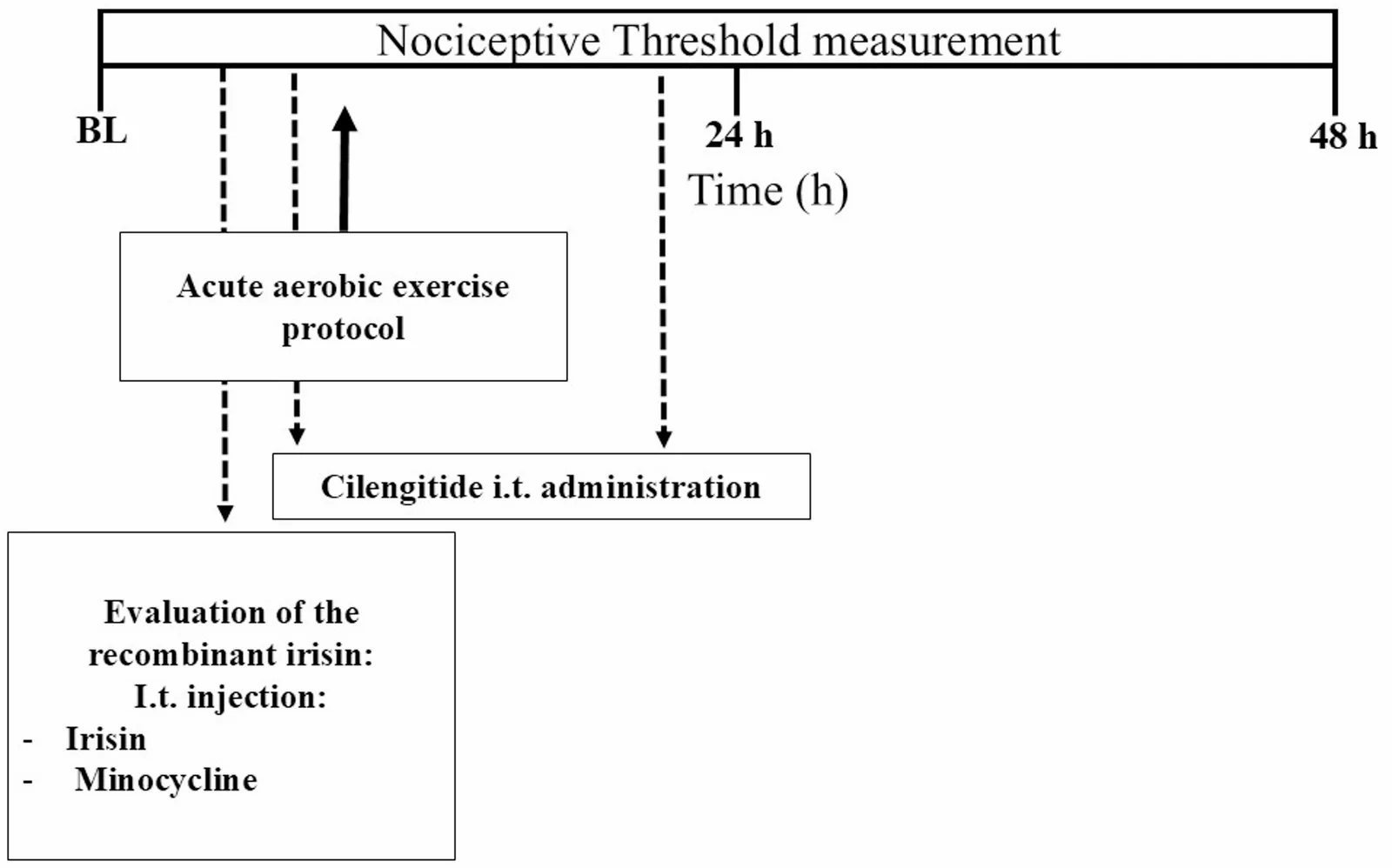
Schematic diagram of the experimental protocol for spinal evaluation of integrin αvβ5 receptor and irisin in DOMS. The baseline latency (BL) of the nociceptive threshold in each mouse was first measured using the von Frey filament test. To investigate the involvement of the spinal integrin αvβ5 receptor, a specific antagonist was administered either 10 min before the onset of the acute progressive exercise or 24 h after its completion. Nociceptive threshold measurements were then repeated at 24 and 48 h post-exercise. The nociceptive effects of irisin were evaluated at 24 and 48 h after its administration. Additionally, the involvement of spinal microglia in this process was assessed by administering minocycline 10 min prior to irisin injection

In experiments involving cilengitide or minocycline, both compounds were administered 10 min prior to the subsequent measurement of the nociceptive threshold (Fig. 1). For experiments assessing the effect of recombinant irisin, the nociceptive threshold was evaluated 24 and 48 h post-administration (Fig. 1).

The number of animals required per group to achieve adequate statistical power was determined based on previous studies conducted in our laboratory (Dos Santos et al. 2020; Elisei et al. 2020). A significance level of 0.05 (corresponding to a Z value of 1.96) was adopted. The estimated standard deviation (σ) was 1.41, and the margin of error (e) was set at 0.22, reflecting variability observed in prior experiments using von Frey filaments ranging from 0.16 g to 2.0 g, with an amplitude of 1.84 g. Considering a relative error of 10%, corresponding to an absolute error of 0.18 g, the calculation yielded a minimum sample size of six animals per group.

### Quantification of cytokine levels

Since cytokines play a key role in modulating the central nociceptive synapse (Zipp et al. 2023), we evaluated the effect of acute aerobic exercise on their levels. Spinal cord tissue samples (segments L4–L6) were collected 24 h after exercise from both exercised and control (non-exercised) mice. Approximately 200 mg of each sample was transferred to a microtube containing 1 ml of PBS and homogenized for 10 min at 3000 rpm at 4 °C. The resulting supernatants were then transferred to 96-well microplates for quantification of IL-1β (#900-K47), TNF-α (#900-K54), and IL-10 (#900-K53) using ELISA kits (PeproTech, Cranbury, NJ, USA), according to the manufacturer’s instructions. The detection limits of the assays were in the low pg/mL range, as specified by the manufacturer. Cytokine levels were measured at 630 nm using a microplate reader (ELX800, BioTek, USA) and analyzed using Gen5 software (BioTek, USA).

### Microglial cell culture and treatment

C8-B4 murine microglial cells (American Type Culture Collection [ATCC], USA) were seeded in 24-well plates at a density of 2 × 10⁵ cells/mL and maintained in Dulbecco’s Modified Eagle’s Medium (DMEM; Sigma-Aldrich, USA) supplemented with 10% fetal bovine serum (Sigma-Aldrich, USA) and 1% penicillin–streptomycin (Life Technologies, USA) at 37 °C in a humidified atmosphere containing 5% CO₂. Experiments were performed in three independent biological replicates (*n* = 3), with each experimental condition assessed in triplicate.

After 48 h of culture, cells were stimulated with recombinant irisin (1 µg/mL) for 6, 12, or 24 h. In additional experiments, cells were treated with lower concentrations of irisin (0.1–0.3 µg/mL) for 24 h. Lipopolysaccharide (LPS; 1 µg/mL for 12 h) was used as a positive control. All treatments were prepared in complete culture medium. At the end of the respective stimulation periods, culture supernatants were collected for cytokine quantification by ELISA, and cells were harvested for flow cytometric analysis.

### Flow cytometric analysis

Microglial activation was assessed by flow cytometry using the Guava easyCyte system (Millipore, MO, USA). Cells were detached with 0.25% trypsin, then resuspended in PBS containing 0.5% bovine serum albumin and 2 mM EDTA. Subsequently, cells were stained with FITC-conjugated anti-CD45 antibody (eBioscience, San Diego, CA, USA) or Annexin V for 30 min at 4 °C. Data were acquired from 10,000 events per sample, and mean fluorescence intensity (MFI) was analyzed using FlowJo software (Tree Star, USA).

### Statistical analysis

Statistical results are presented as mean ± standard error of the mean (SEM). Data normality and homogeneity of variance were assessed using the Shapiro–Wilk and Levene’s tests, respectively. Data were analyzed using either Two-way or One-way ANOVA, followed by Bonferroni’s post-hoc test. A p-value of less than 0.05 was considered statistically significant. All statistical analyses and graph generation were performed using GraphPad Prism 8 software (GraphPad Software, USA). The researchers who conducted the experiments were blinded to treatment allocation, and data were analyzed by an independent investigator unaware of the experimental conditions.

## Results

### Spinal involvement of the integrin αvβ5 receptor in exercise-induced DOMS

**Fig. 2:**
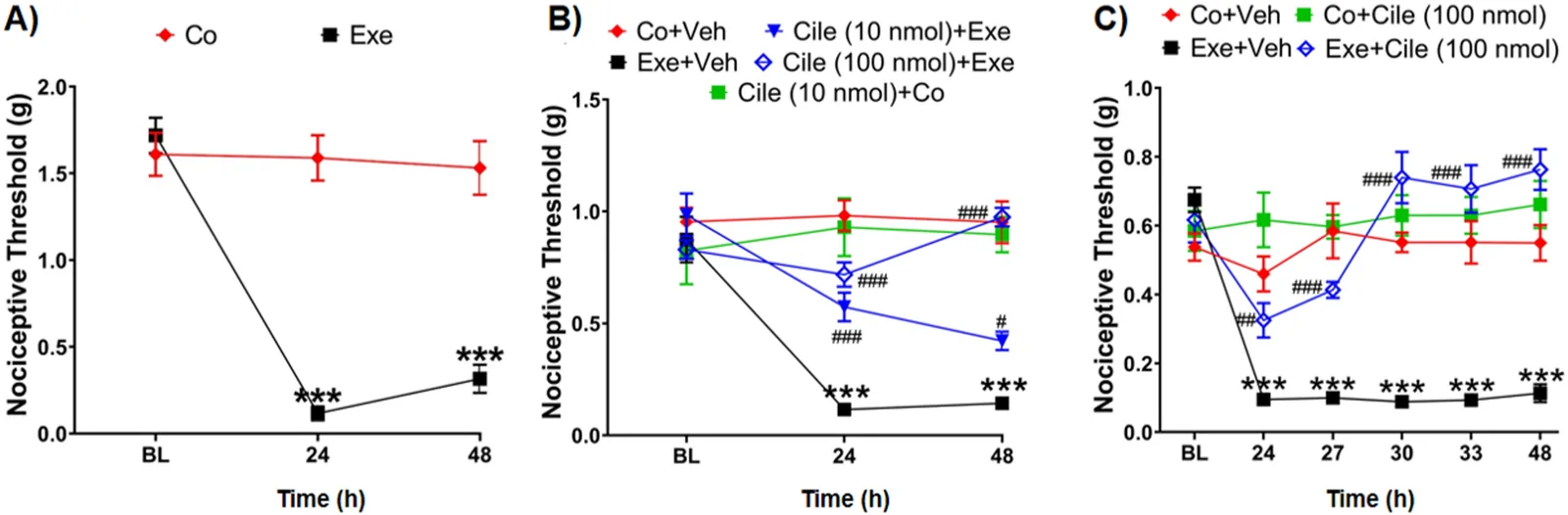
Effect of acute exercise on the development of delayed-onset muscle soreness (**A**) and involvement of spinal integrin αvβ5 receptors, assessed by cilengitide administration before (**B**) or 24 h after exercise (**C**). Panels **A–C** represent independent experiments performed in separate cohorts of animals; therefore, statistical comparisons were performed within each experiment and not across panels. Data are presented as mean ± SEM (*n* = 6 per group). ***p < 0.001 vs. control (Co; non-exercised mice, with or without vehicle [Veh]); #*p* < 0.05 and ###*p* < 0.001 vs. exercised group. Statistical analysis was performed using two-way ANOVA followed by Bonferroni’s post hoc test (factors: time and treatment). BL, baseline; Cile, cilengitide

Following the induction of DOMS via an acute aerobic exercise protocol (Fig. 2A), this study investigated the spinal role of the irisin receptor, integrin αvβ5, in DOMS onset. Intrathecal administration of the integrin αvβ5 antagonist cilengitide significantly reversed DOMS at both 24 h (*p* < 0.001 for 10 and 100 nmol doses) and 48 h (*p* < 0.05 for 10 nmol and *p* < 0.001 for 100 nmol) post-exercise (Fig. 2B). To assess the receptor’s involvement in DOMS maintenance, cilengitide at the highest effective dose (100 nmol) was administered intrathecally 24 h after exercise, resulting in significant reversal of DOMS at 24 (*p* < 0.05), 27, 30, 33, and 48 h (all *p* < 0.001) post-administration (Fig. 2C). Administration of the vehicle (saline) had no effect on the nociceptive threshold. These findings suggest that the irisin receptor integrin αvβ5 contributes to DOMS development and maintenance at the spinal level.

### Irisin contributes to spinal nociception: the role of microglial activation

**Fig. 3:**
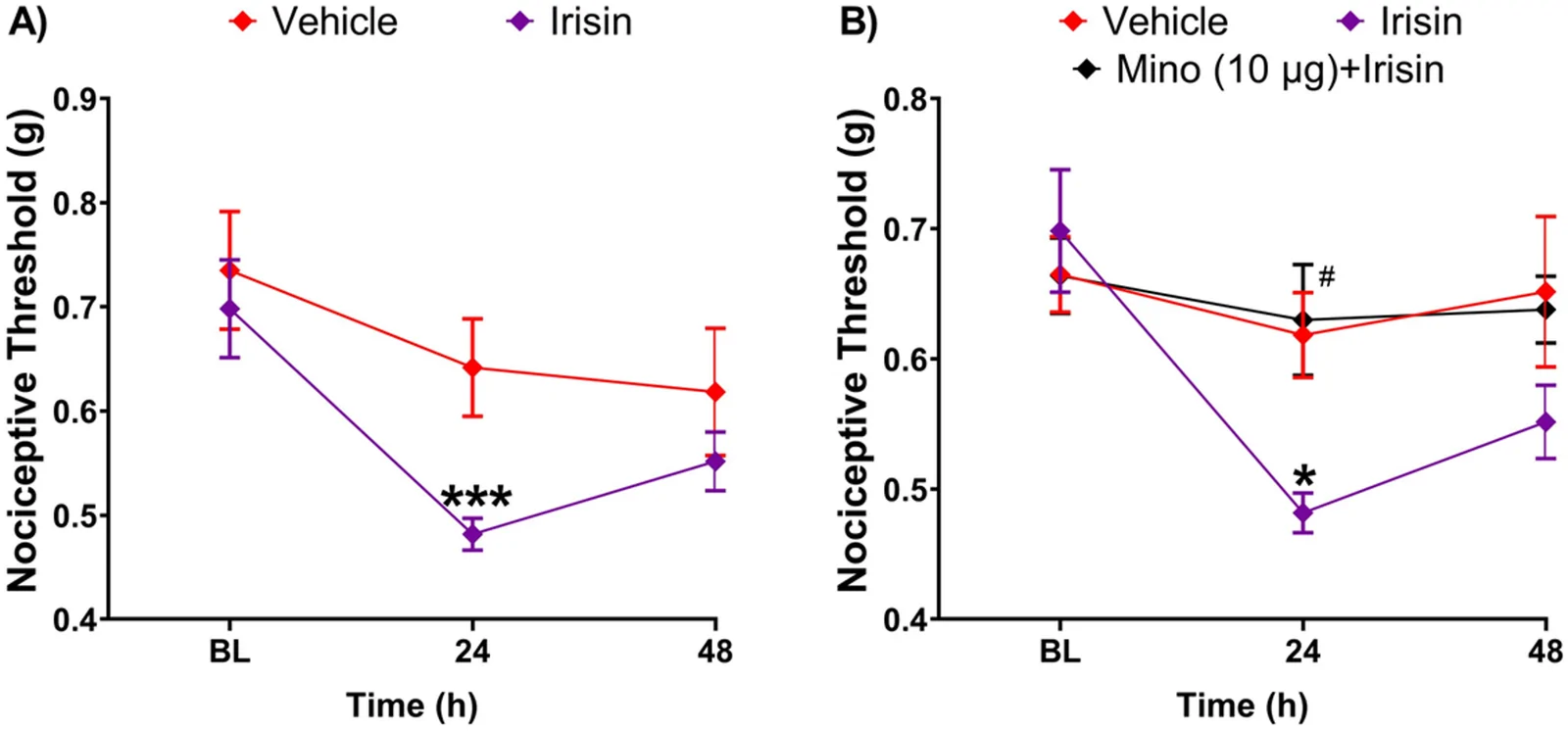
Irisin-induced spinal nociception **(A**) and the involvement of microglia in this process (**B**). Data are presented as mean ± SEM (*n* = 6 per group). *p < 0.05 and ***p < 0.001 vs. control (non-exercised mice); #*p* < 0.05 vs. irisin-treated group. Statistical analysis was performed using two-way ANOVA followed by Bonferroni’s post hoc test (factors: time and treatment). BL, baseline latency; Mino, minocycline

Following our previous findings that suggesting the involvement of the irisin integrin αvβ5 receptor in DOMS, we investigated whether irisin itself induces nociception. As shown in Fig. 3A, intrathecal administration of irisin (250 ng) resulted in a significant reduction in the nociceptive threshold 24 h post-administration (*p* < 0.05).

Considering that activated microglia may contribute to pain sensitization at the spinal level (Gazerani 2021), the next phase of this study aimed to evaluate the involvement of microglia in irisin-induced nociception. The results indicated that intrathecal administration of the microglial inhibitor minocycline significantly reversed (*p* < 0.05) the nociceptive effect induced by irisin (Fig. 3B).

Microglia can exhibit the pro-inflammatory M1 phenotype, which plays a key role in spinal nociceptive processing, or the anti-inflammatory M2 phenotype, which is involved in antinociceptive mechanisms (Guo et al. 2022). Cytokines are among the primary mediators released by microglia that modulate nociceptive synapses. Therefore, the present study evaluated spinal levels of pro-inflammatory cytokines, including TNF-α and IL-6, as well as the anti-inflammatory cytokine IL-10 during DOMS, alongside the involvement of the integrin αvβ5 receptor in this process.

**Fig. 4:**
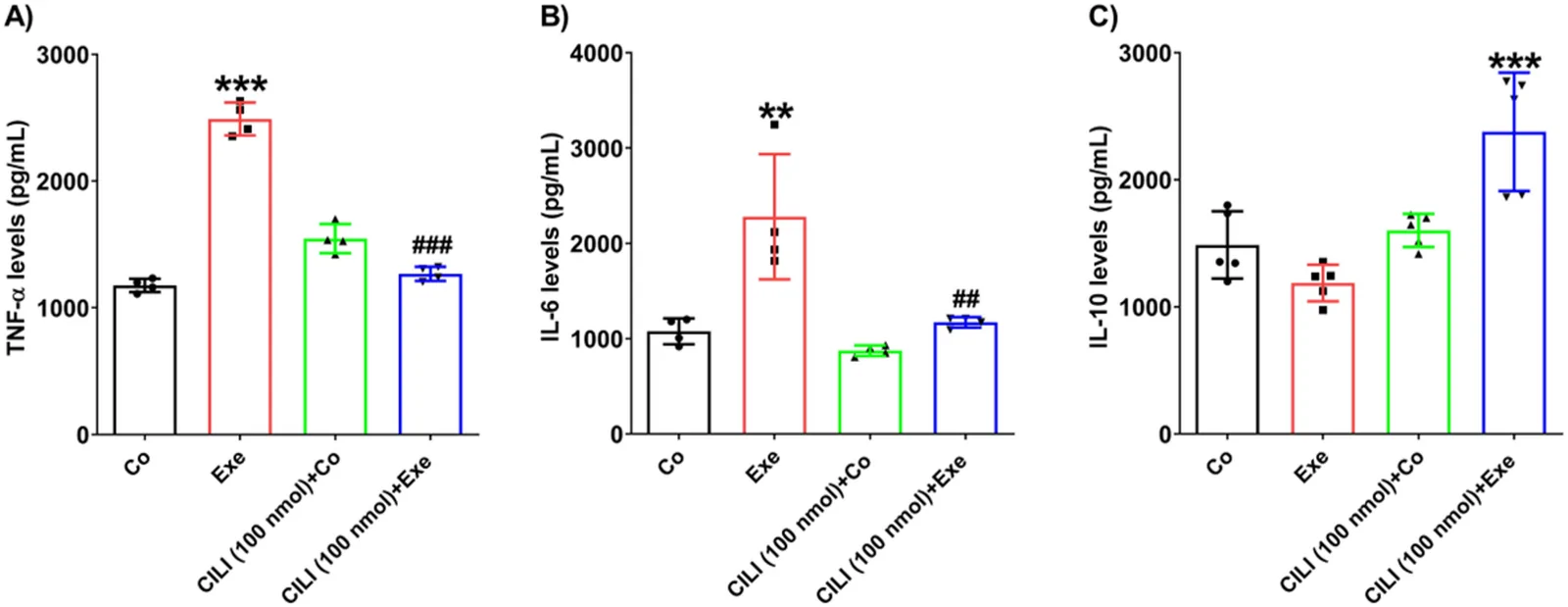
Involvement of the integrin αvβ5 receptor in spinal levels of IL-1β (**A**), TNF-α (**B**), and IL-10 (**C**) during DOMS. Data are presented as mean ± SEM (*n* = 5 per group). **p < 0.01 and ***p < 0.001 vs. control (Co; non-exercised mice); ##*p* < 0.01 and ###*p* < 0.001 vs. exercised group (Exe). Statistical analysis was performed using one-way ANOVA followed by Bonferroni’s post hoc test. Exe, exercise group; Co, control group; Cile, cilengitide

Spinal levels of TNF-α and IL-6 were significantly increased 24 h after exercise (*p* < 0.01 and *p* < 0.001, respectively), and this elevation was prevented by intrathecal administration of cilengitide (*p* < 0.01 and *p* < 0.001) (Fig. 4A and B). Furthermore, cilengitide significantly increased levels of the anti-inflammatory cytokine IL-10 (*p* < 0.001) (Fig. 4C).

### Irisin induces microglial activation accompanied by cytokine release

**Fig. 5:**
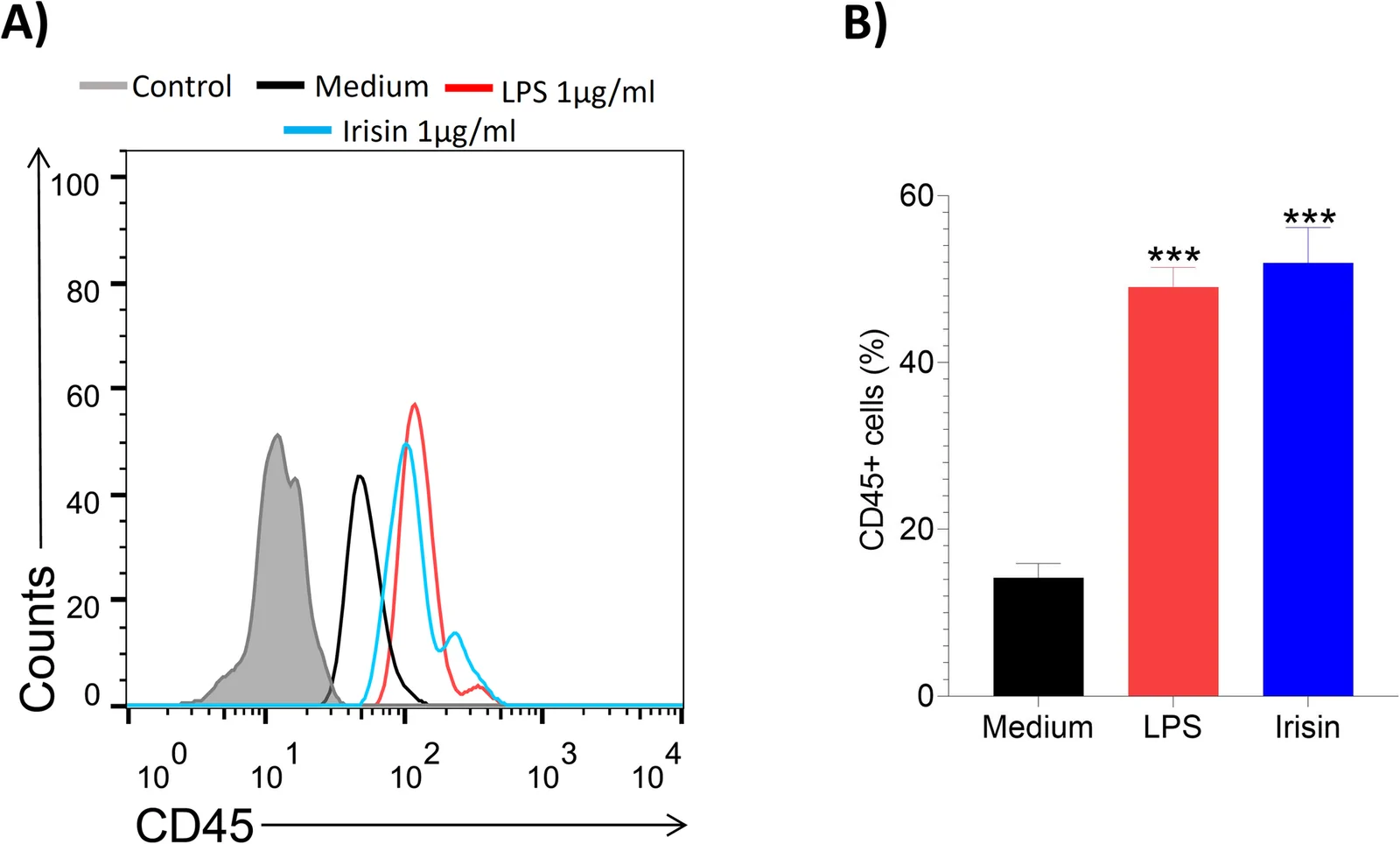
Effect of irisin on microglial activation. (**A**) Representative flow cytometry histograms showing CD45 expression in C8-B4 microglial cells. The gray curve represents the unstained cell control, used to establish background/autofluorescence, whereas the black curve represents untreated cells maintained in complete culture medium and stained for CD45, indicating basal CD45 expression. The light blue curve represents cells stimulated with recombinant irisin (1 µg/mL for 12 h), and the red curve represents cells stimulated with LPS (1 µg/mL for 12 h), used as a positive control. (**B**) Quantification of CD45 expression in untreated cells and following stimulation with irisin or LPS. Data are presented as mean ± SEM from three independent experiments, each performed in triplicate. ***p < 0.001 vs. untreated control (complete culture medium). Statistical analysis was performed using one-way ANOVA followed by Bonferroni’s post hoc test

**Fig. 6:**
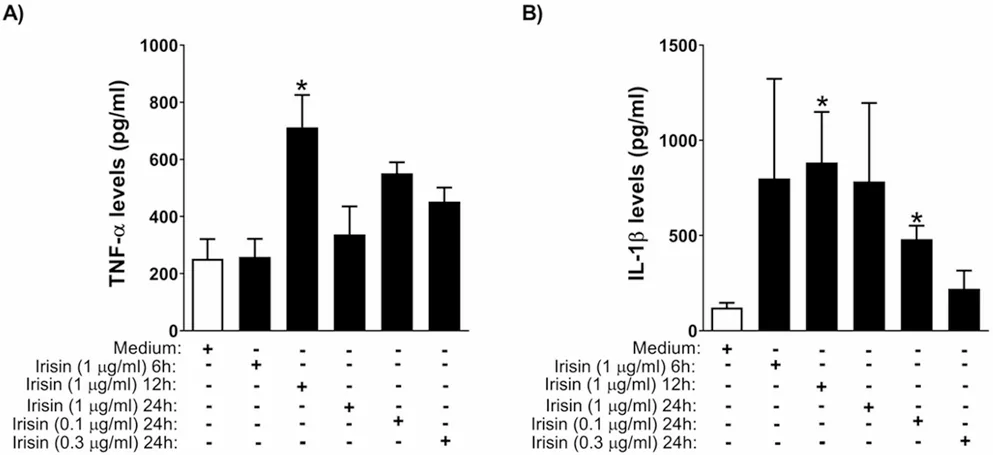
Irisin increases pro-inflammatory cytokine production in microglial culture supernatants. Irisin treatment significantly increased TNF-α (**A)** and IL-1β (**B**) levels in the culture supernatant. Data are presented as mean ± SEM. *p < 0.05 vs. untreated control (medium only). Statistical analysis was performed using one-way ANOVA followed by Bonferroni’s post hoc test

In vitro, stimulation with recombinant irisin (1 µg/mL) for 12 h significantly increased microglial activation, with an effect comparable to that induced by LPS (1 µg/mL for 12 h), which was used as a positive control (Fig. 5A and B). In addition, irisin treatment significantly increased the levels of the pro-inflammatory cytokines TNF-α and IL-1β in the culture supernatant. Significant increases were observed following 12 h of stimulation with 1 µg/mL irisin (*p* < 0.05) and following 24 h of stimulation with lower irisin concentrations (0.1 and 0.3 µg/mL; *p* < 0.05) (Fig. 6A and B).

## Discussion

The present study demonstrated that the irisin receptor integrin αvβ5 is involved in the development of DOMS at the spinal level. Moreover, the findings indicate that irisin can induce nociception through spinal mechanisms that involve microglial activation.

Irisin, a secreted protein involved in the regulation of metabolism and tissue stress, exerts its physiological effects primarily through interaction with the integrin αVβ5 receptor (A et al., 2023). Although the integrin αVβ3 receptor is among the earliest studied integrins and is known to bind various ligands—including vitronectin, TGF-β, fibronectin, osteopontin, neural cell adhesion molecule L1, fibrinogen, von Willebrand factor, thrombospondin, fibrillin, and tenascin (Pang et al. 2023) — few studies have demonstrated its affinity for irisin (Liu et al. 2022).

To our knowledge, the present study is the first to evaluate the role of irisin and its receptor in the context of DOMS. Moreover, few studies have investigated this signaling pathway in pain. Interestingly, in contrast to our findings, two previous studies have reported antinociceptive effects of irisin. One study demonstrated that systemic administration of irisin (i.p.) reversed hyperalgesia in an acute inflammatory pain model induced by intraplantar injection of Freund’s adjuvant in mice (Rahman et al. 2024). Another study reported a reversal of mechanical allodynia in rats with neuropathic pain following intrathecal administration of irisin (Dameni et al. 2018). We hypothesize that the effects of irisin are dose-dependent, as studies administering higher doses—either intrathecally or intraperitoneally—have observed antinociceptive outcomes. In contrast, the present study employed a much lower dose, at which a pronociceptive effect was observed. Additionally, factors such as the pain model used, the animal strain, and the type of recombinant irisin may also influence the observed effects. Nevertheless, further research is required to confirm these hypotheses.

Regarding the integrin αVβ5 receptor, which our results suggest plays a spinal role in DOMS, there are currently no studies investigating this receptor in pain. However, given that αVβ5 can be activated by numerous ligands, as previously described, irisin emerges as a strong candidate in the context of exercise-induced DOMS. This is supported by evidence showing that irisin is robustly released during exercise (Parada-Sánchez et al. 2022; Mohammad Rahimi et al. 2022). In addition, our results demonstrated that the integrin αVβ5 receptor may be involved in DOMS development both at the onset of exercise and during the post-exercise period, specifically 24 h later. Previous studies have shown that the rapid increase in irisin concentration immediately after exercise onset may result from the proteolytic cleavage of fibronectin type III domain-containing protein 5 (FNDC5), which is localized on the plasma membrane of muscle cells (Tommasini et al. 2024). This cleavage leads to the release of irisin into the bloodstream in response to exercise. Furthermore, de novo FNDC5 synthesis, induced by activation of peroxisome proliferator-activated receptor gamma coactivator 1-alpha (PGC1-α), may occur hours after exercise cessation (Tommasini et al. 2024). These mechanisms may explain why irisin production by muscle increases both during and after acute aerobic exercise, potentially activating the integrin αVβ5 receptor and thereby contributing to the development and maintenance of DOMS.

The present study also demonstrated microglial involvement in irisin-induced nociception. Given the established role of microglia in central pain sensitization, we hypothesize that irisin contributes to microglial activation, which may underlie the maintenance of DOMS. Supporting this, pretreatment with the microglial inhibitor minocycline effectively blocked the nociceptive effects induced by intrathecal administration of irisin. Consistent with this, previous studies have shown that exercise-induced DOMS is dependent on spinal neuroinflammation, characterized by microglial and astrocyte activation, increased pro-inflammatory cytokine production, and NF-κB signaling, all of which contribute to nociceptive sensitization (Borghi et al. 2022).

Most of the evidence in the literature highlights irisin’s role as a neuroprotector and modulator of inflammation in various pathologies (Sandoval and Gómez 2024). Given that delayed onset muscle soreness (DOMS) can serve as a protective mechanism to prevent further injury—particularly following unaccustomed exercise (Cheung et al. 2003) — irisin may play a key role within this response. Although previous studies have demonstrated irisin’s antinociceptive and anti-inflammatory effects, including inhibition of the pro-inflammatory microglial M1 phenotype (Dameni et al. 2018; Rahman et al. 2024; Li et al. 2025), we hypothesize that the pro-nociceptive effect observed in the present study reflects irisin’s complex neuroprotective and neuromodulatory functions during the acute phase. It is likely that following a period of aerobic training, irisin’s effects will shift toward its classically described anti-inflammatory and antinociceptive roles.

Furthermore, both irisin and the integrin αVβ5 receptor are expressed in microglia (Welser-Alves et al. 2011; Wang et al. 2022a, b; Kim et al. 2023). Therefore, we propose that during acute aerobic exercise, increased irisin levels may activate the integrin αVβ5 receptor expressed on spinal microglia, leading to the release of pro-inflammatory cytokines that contribute to modulation of the nociceptive synapse. Our results demonstrated a significant increase in the cytokines IL-6 and TNF-α 24 h after exercise. Interestingly, this increase was prevented in the group pre-treated with the integrin αVβ5 receptor antagonist cilengitide. IL-6 and TNF-α sensitize second-order nociceptive neurons by activating specific receptors, which trigger intracellular signaling cascades. These cascades lead to the phosphorylation of ion channels and glutamate receptors, thereby facilitating the transmission of nociceptive signals (Boadas-Vaelloet al., 2016). Interestingly, we found that cilengitide increases the levels of the anti-inflammatory cytokine IL-10, which exerts effects opposite to those previously described by hyperpolarizing second-order neurons (Boadas-Vaelloet al., 2016). These data further reinforce that the integrin αVβ5 receptor is involved in the modulation of the neuroinflammatory response that contributes to DOMS.

This study also demonstrated that irisin-induced microglial activation in vitro leads to increased levels of the pro-inflammatory cytokines TNF-α and IL-1β. In most previous studies, the effects of irisin on microglia were evaluated either in in vivo models of neuroinflammation or in vitro following pre-insult stimulation of the cells. These studies reported that treatment with irisin at doses much lower than those used in the present study reversed microglial activation (Jin et al. 2019; Wang et al. 2022a, b, 2024). As previously discussed, although these findings differ from those reported in the literature, factors such as the experimental model, stimulation duration, irisin concentration, and isoform may influence the results. Moreover, these findings not only reinforce our previous data but also highlight the importance of further investigations into the neuroinflammatory and nociceptive roles of the irisin/integrin αVβ5 receptor pathway.

We agree, however, that the origin of irisin and its ability to reach the central nervous system are critical aspects that warrant further discussion. Irisin is predominantly released from skeletal muscle during exercise (Boström et al. 2012), and accumulating evidence suggests that circulating irisin can cross the blood–brain barrier and exert central effects, including modulation of neuroinflammation and synaptic function (Wrann et al. 2013; Lourenco et al. 2019). In addition, αvβ5 integrin has been identified as a functional receptor for irisin (Kim et al. 2019), and is expressed in central nervous system cells, including microglia, supporting the biological plausibility of this signaling pathway.

Several limitations should be considered when interpreting the present findings. First, all experiments were conducted exclusively in male mice. Although this experimental design was adopted to reduce hormonal variability, it may limit the generalizability of our findings. Sex is an important biological variable influencing nociceptive processing, immune responses, and neuroimmune communication (Vacca et al.,^2021^; Barcelon et al., 2023). Therefore, future studies including female mice are needed to determine whether the involvement of αvβ5 integrin signaling and microglia in DOMS exhibits sex-dependent differences.

A second limitation concerns the behavioral assessment of mechanical hypersensitivity. Plantar von Frey testing does not directly measure primary deep-muscle mechanical hyperalgesia, as the mechanical stimulus is applied to the plantar surface rather than to the exercised gastrocnemius muscle. Instead, the observed plantar mechanical hypersensitivity may reflect, at least in part, secondary mechanical hyperalgesia associated with increased nociceptive input from the exercised muscle and subsequent sensitization of spinal nociceptive circuits. Importantly, primary muscle mechanical hyperalgesia induced by the same treadmill exercise protocol has previously been demonstrated by our group. Dos Santos et al. (2020) directly assessed mechanical sensitivity over the gastrocnemius muscle using a pressure application measurement (PAM) device and reported a significant reduction in the muscle mechanical nociceptive threshold following exercise. Moreover, previous evidence of spinal neuroimmune activation in this model supports the possibility that central sensitization contributes to the plantar mechanical hypersensitivity observed in the present study. Accordingly, our behavioral findings should be interpreted as exercise-induced plantar mechanical hypersensitivity rather than as a direct measurement of primary deep-muscle hyperalgesia.

Another important limitation relates to the interpretation of irisin signaling. The present study relied on exogenous intrathecal administration of irisin to investigate its central effects, rather than directly assessing the contribution of endogenous exercise-derived irisin. Although our findings support the ability of exogenous irisin to modulate spinal nociceptive responses and microglial activation through αvβ5 integrin-dependent mechanisms, they do not establish a direct causal relationship between peripheral irisin release and the central effects observed during DOMS. Furthermore, endogenous irisin concentrations were not quantified in skeletal muscle, plasma, or spinal cord, and the systemic consequences of αvβ5 integrin antagonism were not evaluated. Because αvβ5 integrin interacts with multiple endogenous ligands, the observed effects cannot be attributed exclusively to irisin signaling, and the potential contribution of alternative ligand-dependent pathways cannot be excluded. Although circulating irisin has been reported to cross the blood–brain barrier, the extent, kinetics, and physiological relevance of this transport remain incompletely understood.

Finally, the use of CD45 as an activation-associated marker does not fully characterize the complexity and heterogeneity of microglial activation states. Future investigations incorporating complementary microglial markers, such as Iba1 and TMEM119, together with the quantification of endogenous irisin in peripheral tissues and the central nervous system, will be important to clarify the physiological relevance of this pathway. In addition, more selective genetic or pharmacological approaches targeting αvβ5 integrin signaling, combined with experiments designed to distinguish the contributions of endogenous irisin from those of other integrin ligands, may help establish whether exercise-derived irisin contributes causally to spinal neuroimmune responses during DOMS.

In conclusion, our findings suggest that spinal αvβ5 integrin signaling and microglia contribute to the nociceptive responses associated with DOMS. Furthermore, the effects of exogenously administered irisin support a potential role for irisin-sensitive αvβ5 integrin signaling in modulating spinal nociception and microglial activation. However, the contribution of endogenous exercise-derived irisin to these mechanisms remains to be established. These findings provide further insight into the neuroimmune mechanisms associated with DOMS and may guide future investigations aimed at identifying potential therapeutic targets.

## Data Availability

No datasets were generated or analysed during the current study.
